# Importance of Capillary Leak and Nocturia in Defining and Successfully Treating Idiopathic Edema

**DOI:** 10.3390/jcm14217625

**Published:** 2025-10-27

**Authors:** John K. Maesaka, Louis J. Imbriano, Candace Grant, Minesh Khatri, Nobuyuki Miyawaki

**Affiliations:** Division of Nephrology and Hypertension, Department of Medicine, NYU Langone Hospital Long Island, NYU Grossman Long Island School of Medicine, Mineola, NY 11501, USA

**Keywords:** cyclical edema, hydrostatic pressure, oncotic pressure, nocturia, weight gain

## Abstract

**Background/Objectives:** Idiopathic edema (IE) in women is characterized by swelling of hands and face followed by increasing abdominal and truncal girth, bloating, edema, >1.4 kg weight gain when in upright posture, and nocturia that eliminates the retained fluid. A capillary leak is the primary pathophysiologic abnormality that induces different clinical presentations that were considered untreatable. **Methods:** We utilized different aspects of Starling forces of edema formation and treated four uncomplicated cases of IE by reducing salt intake with or without diuretics and two cases of life-threatening cases due to seizures and coma induced by acute hyponatremia in one and postural dizziness, fainting, and fractures and dislocations of joints in another. **Results:** All four uncomplicated cases of IE were treated by reducing salt intake with or without diuretics that eliminated the weight gain and nocturia. The patient with hyponatremia never developed hyponatremia by reducing water intake and signs and symptoms of IE by salt restriction and diuretic therapy and eliminated the postural hypotension, falls, and fainting by use of support hose that increased interstitial hydrostatic pressure to eliminate fluid shifting from intravascular to interstitial spaces. **Conclusions:** A leaky capillary induces pathophysiologic changes that activate different metabolic pathways. IE is now a treatable condition, following: 1. Salt restriction with or without diuretics for the cyclical weight gain, and 2. Water restriction for hyponatremia, hyponatremic seizures, and coma and 3. support hose for postural hypotension, postural dizziness, and fainting. IE is unrecognized and probably more common than it is perceived.

## 1. Introduction

Idiopathic edema (IE) is a disease of unknown etiology that occurs virtually exclusively in women between the ages of 20 and 60 years. It is characterized by a weight gain of >1.4 kg from morning to evening mostly in obese women with variable degrees of non-pitting edema of face and hands on awakening in the morning that are unrelated to menses. This is followed by increased abdominal girth with expansion of waist size, bloating, swelling of the trunks with tightening of the shirt, and non-pitting edema of the lower extremities after assuming an upright posture. Once in bed, they eliminate the fluid retained during the day in the form of nocturia every 2 to 3 h to maintain stable weights the following morning [1,2,3,4,5,6]. The failure to include nocturia in characterizations of IE implies that there is no escape from the daily increase in weight. The daily weight gain without an escape would lead to an unabated increase in fluid overload that would lead to increasing edema and possibly congestive heart failure (CHF). The diagnosis of IE should be considered especially in any woman complaining of nocturia as an essential feature and an early clue to its diagnosis. Lack of nocturia would rule out the diagnosis of IE.

Various escape phenomena have been well characterized that are similar to the nocturia, preventing perpetual fluid overload in IE [7]. Examples include deoxycorticosterone acetate (DOCA) escape where the body counteracts the sodium-retaining properties of DOCA by activating humoral, hemodynamic, and neural mechanisms to reach an equilibrated state where sodium output matches input but at a higher extracellular volume [8]; pitressin (vasopressin) escape in the syndrome of inappropriate secretion of antidiuretic hormone as its V2 receptor down regulation reduces the distal tubule water reabsorption [9]; diuretic escape from chronic diuretic administration such as furosemide by hypertrophy and proliferation of sodium transporters in the distal nephron [10]; renal salt wasting escape from the recently identified natriuretic protein, haptoglobin-related protein without signal peptide, where physiologic adjustments such as that found in DOCA escape will eliminate the sodium wasting by reaching an equilibrated state when sodium intake matches sodium output but at a lower blood volume [11,12]; and now IE escape [7,13]. A deficiency of a functional escape system can lead to serious hemodynamic consequences such as the persistent conservation of sodium in CHF, persistent sodium wasting in severe Addison’s disease, or profound capillary leak in IE where intravascular volume depletion can be life-threatening [14]. Patients with IE have also been characterized by obesity, diabetes with frequent miscarriages, having large babies, and being significantly irritable and emotionally unstable, Table 1 [1,2].

Increased morbidity is not associated with increased mortality [3,4]. The multitude of nomenclatures such as cyclical edema, periodic edema, fluid retention syndrome, capillary leak syndrome and orthostatic edema reflects a state of confusion that, until recently, lacked a unified understanding of the pathophysiology and successful treatment of this enigmatic disease [13,14]. This poorly defined condition has not been fully recognized as a distinct clinical entity. It is also considered to be an untreatable condition. We sorted through some classic studies which set the pathophysiologic foundation to base our successful treatment of the varied clinical presentations of IE, including those that are life-threatening [5,6,13,14].

This review focuses on capillary leak as the single underlying physiologic abnormality leading to the complex physiology of edema formation via Starling’s forces [15]. We demonstrate how a variable increase in capillary leak can induce clinical presentations that appear to be cyclical, disconnected and potentially life-threatening. Addressing the metabolic pathways that are affected by a capillary leak led to successful treatment of the diverse clinical presentations of IE with significant improvement in physical and emotional quality of life, Table 2 [5,6,13,14,15].

## 2. Starling Forces That Contribute to Edema Formation

According to Starling’s forces, the movement of fluid between the intravascular and interstitial spaces is largely dependent on differences in hydrostatic and oncotic pressures between both spaces as well as the capillary membrane permeability [16]. Pre-capillary arteriolar smooth muscle autoregulation prevents an increase in venous hydrostatic pressure to explain the absence of edema in hypertensive patients. An increase in intravascular hydrostatic pressure as seen in CHF will move fluid from intravascular to interstitial spaces or conversely move fluid from interstitial to intravascular spaces if interstitial hydrostatic exceeds intravascular hydrostatic pressure. Differences in oncotic pressure between intravascular and interstitial spaces will also determine the net fluid movement between these spaces. When intravascular oncotic pressure decreases because of a loss of proteins in the urine as seen in patients with nephrotic syndrome, the net movement of intravascular fluid to the interstitial spaces leads to edema formation. Under normal circumstances, interstitial oncotic pressure is much lower than that of plasma because the vascular membrane is impermeable to larger protein molecules.

Conditions which increase vascular membrane permeability will increase the interstitial oncotic pressure as larger proteins enter the interstitium with water to increase edema formation. The lymphatic system also drains the interstitial fluid [16]. As will be discussed below, the increase in capillary leak has been documented, but the mechanisms for this isolated phenomenon have yet to be identified.

## 3. Edema Formation in Idiopathic Edema, Evidence for Capillary Leak, and Its Pathophysiologic Consequences

**Figure 1:**
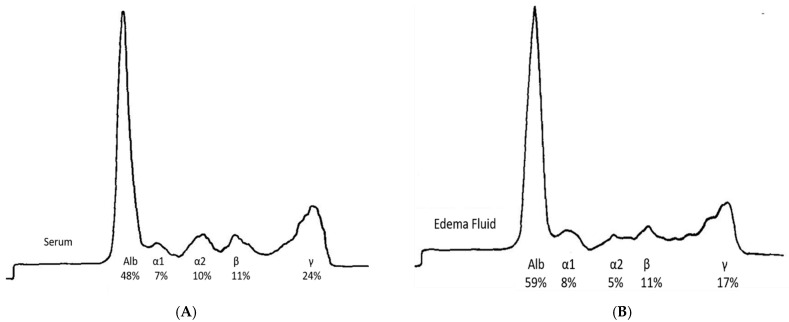
(**A**) Electrophoretic pattern of normal serum; (**B**) Electrophoretic pattern of edema fluid in IE. Adapted from M. Coleman, et al. [15].

Prior explanations of IE emphasized diuretic abuse and refeeding after a period of fasting such that the well-defined presence of capillary leak was essentially overlooked [17,18]. Several studies provide evidence on how the physiologic consequences of an increase in capillary leak can lead to life-threatening consequences [14,19]. Studies indicate that the protein content, and hence, oncotic pressure in the interstitial fluid, is increased in IE patients unlike patients with edema due to CHF, cirrhosis, or nephrotic syndrome. As noted in Figure 1A,B, an electrophoretic pattern of the interstitial edema fluid from an IE patient was very similar to that of normal plasma with proteins as large as 200 kDa being present [15]. The nearly identical patterns suggest the vascular membrane in IE is very leaky, allowing larger proteins to move from intravascular to interstitial spaces.

**Figure 2:**
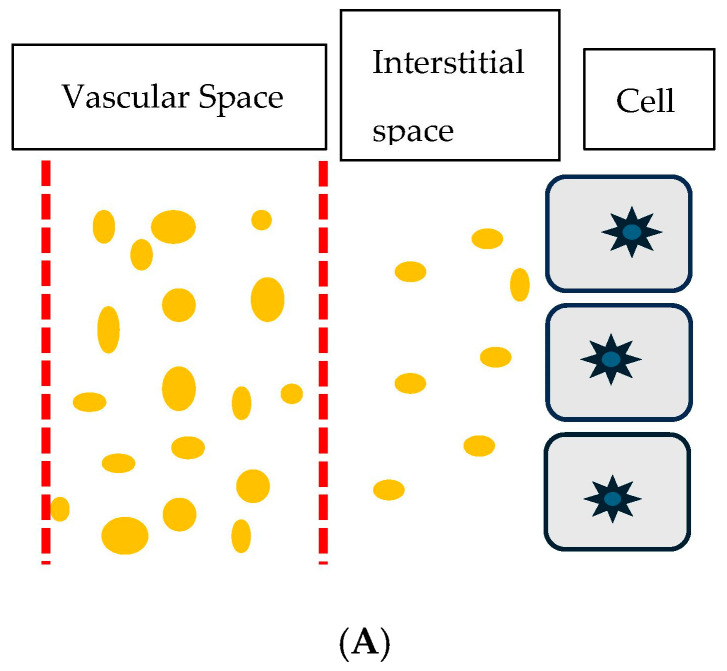

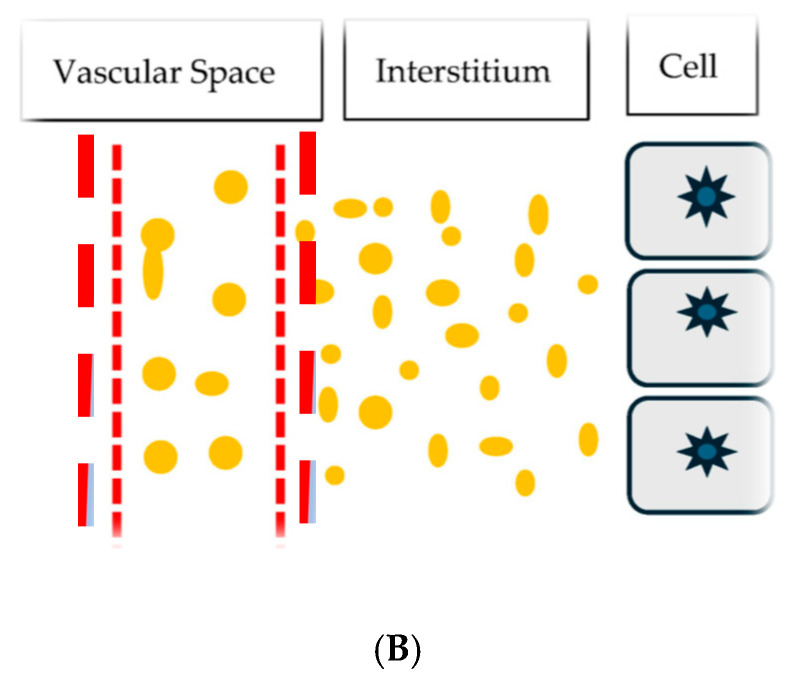
(**A**) Cartoon showing normal vascular membrane depicted as vertical red hashed lines, intravascular volume as distance between vertical lines, spaces in the hashed lines representing holes in vascular membrane, dots of varying sizes representing molecular weights of protein and distance between vertical hashed line on right and cells with nuclei representing interstitial space and volume. Small dots in interstitial space represent small protein molecules able to move through small holes in vascular membrane for protein to move from intravascular to interstitial space. (**B**) Cartoon showing larger space between hashed vertical lines representing larger holes in vascular membrane to permit larger dots or larger protein molecules and fluid to move from intravascular to interstitial space. This increases the amount of larger dots or larger protein molecules and fluid to move into the interstitial space to increase the distance or volume between the vertical hashed line on right and cells with nuclei and decrease intravascular volume between vertical hashed lines.

The protein content of 3.1 gm/dL in the interstitial edema fluid of the IE patient was significantly higher than the 0.1 to 0.3 gm/dL in the edema fluid in cirrhosis of the liver, 0.39 gm/dL in CHF, and 0.1 to 0.11 gm/dL in nephrotic syndrome, Figure 2A,B [16]. The presence of large proteins in the interstitial fluid of IE patients suggests that an increase in membrane permeability or capillary leak is responsible for the presence of high molecular weight proteins and high protein content in interstitial fluid. This results in fluid movement and the activation of various physiologic pathways (described in Table 2) and depicted in Figure 2A,B [6,16].

When IE patients assume an upright position as on a tilt table, there is an increase in hemoglobin and hematocrit and decrease in blood volume with an increase in edema formation that suggest an increase in fluid moving from intravascular to interstitial spaces. The reduced blood volume reduces glomerular filtration rate (GFR), increases plasma renin, aldosterone, and antidiuretic hormone (ADH) and decrease urinary volume and solute excretion that reflect the decrease in intravascular volume. It is in this upright position that the patient experiences truncal and abdominal tightness, increase in waist size, bloating, and edema, see Table 2 [6].

When the subject assumes a recumbent position at bedtime, the interstitial edema fluid shifts rapidly into the intravascular space to increase blood volume and decrease hemoglobin, hematocrit, renin, aldosterone, ADH, and GFR to collectively increase solute and water excretion in the form of nocturia to restore stable edema-free weights the following morning, see Table 2 [6]. The cyclical development of edema with nocturia highlights the quintessential role of capillary leak in IE [16]. The absence of a cyclical nature of edema formation in conditions with intact vascular membrane permeability such as in cirrhosis, heart failure, and nephrosis designates capillary leak as the major contributor to the cyclical nature of edema formation in IE.

## 4. Successful Treatment of Idiopathic Edema, Including Life-Threatening Complications

**Figure 3:**
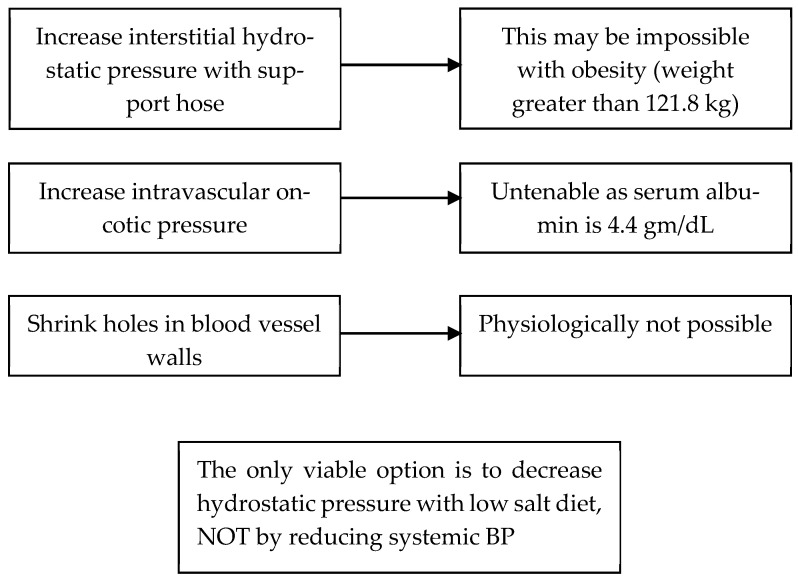
Options to treat a patient with IE according to Starling’s forces.

The treatment of IE has not been well defined and has been considered to be an untreatable disease. We treated four patients who met the criteria for IE by addressing the factors that contributed to edema formation according to Starling’s forces [13]. We reasoned through each variable and concluded that we could not increase intravascular oncotic pressure by increasing plasma protein content, lowering systemic blood pressure to reduce hydrostatic pressure in the veins or eliminate the capillary leak, Figure 3. We were left with two potential therapeutic options; 1. increase interstitial hydrostatic pressure to reduce or prevent proteins and fluid from leaving the intravascular space or 2. reduce intravascular hydrostatic pressure by reducing sodium intake. The first option to increase interstitial hydrostatic pressure could be accomplished by wearing support hose that extended up to the waist or higher. All four patients had body mass indices in the maximally obese ranges that made this option impossible to execute. The only viable option was to reduce intravascular hydrostatic pressure by reducing sodium intake, Figure 3 [13].

According to the American Heart Association website, a low salt diet is to ingest < 2000 mg of sodium per day, which is equivalent to ingesting 5 g of salt. Every patient was seen without recruitment as part of the group’s outpatient practice. When it became apparent that these patients were treatable by methods described in this manuscript, we obtained approval from the internal review board for human research to include them in future publications. All patients had input from a dietitian to estimate their sodium intake and adherence to a low sodium diet was readily evident by an increase in truncal and abdominal girth, edema, weight gain, and nocturia. A low sodium diet was encouraged but a higher sodium intake was permissible to improve quality of life. At least two patients liberalized their sodium intake and took 20 mg of furosemide every morning to avoid the 3–5 days of discomfort. Others only took 20 mg of furosemide when they had a meal that was high in sodium the night before. In the end, each patient was given the option to take 20 mg of furosemide every morning while maintaining a high sodium diet.

**Figure 4:**
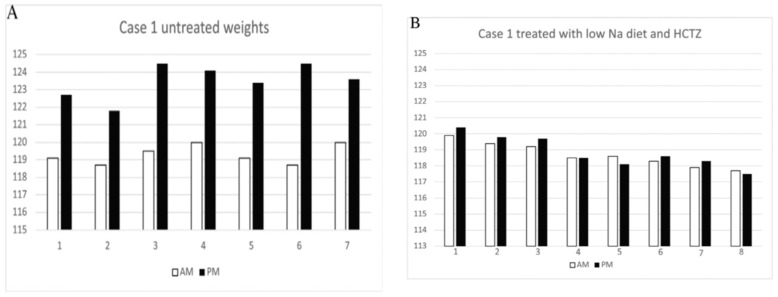
(**A**) Weights in kg taken in AM and PM for 7 days while on a daily sodium intake of 3 to 4 g with nocturia × 4. Patient experienced increasing bloating and edema formation during the day and was free of edema due to the nocturia on arising the following morning. (**B**) Weights in kg in AM and PM for 7 days while on a daily sodium intake of 1 g and hydrochlorothiazide with nocturia × 1. Low sodium diet with HCTZ prevented edema formation and weight gain with reduction in nocturia. Adapted from K. Nayyar, et al. [13].

The first patient was a 44-year-old woman with a body weight of 121.8 kg, hypertension, polycystic ovary syndrome (PCOS), type 2 diabetes mellitus, antiphospholipid syndrome with multiple miscarriages, sleep apnea, and hypercholesterolemia who presented with typical symptoms of IE for 10 years. Utilization of support hose that extended up to her thorax was previously abandoned because of the difficulty in being fitted, the discomfort of having this cumbersome garment on during most of the day while being in an upright position, and appearance of edema above the garment in the chest. She gained 3.5 to 5.5 kg from morning to bedtime while on a daily sodium intake of 3 to 4 gm/day with nocturia × 5 and stable edema-free weights the following morning, Figure 4A. We gradually reduced her sodium intake to 1 g/day, which yielded weights that did not differ from her morning weights by more than 1 kg with a reduction in nocturia to 1, Figure 4B [13].

The second patient was a 56-year-old obese woman with a history of PCOS, hypertension, gastro-esophageal reflux, primary hyperparathyroidism with parathyroidectomy, total hysterectomy with bilateral oophorectomy, and hypercholesterolemia, who presented with signs and symptoms that met the criteria for the diagnosis of IE for 5 years. She weighed 95 kg and had weight gains between 1.4 to 2.3 kg between morning and bedtime with nocturia every 2 to 3 h. She gained less than 0.7 kg during the day after reducing her sodium intake to approximately 1.0 g/day.

The third patient was a 46-year-old woman with a history of type 2 diabetes mellitus, proteinuria, hypertension, hypercholesterolemia, PCOS, biopsy diagnosis of focal segmental glomerulonephritis of the obesity type, and obesity weighing 127.7 kg. The diagnosis of IE was entertained and proven because of history of nocturia × 3 to 4 over the previous 4 years by morning to bedtime weight gains of 1.8 to 2.7 kg. She denied swelling of face or hands on arising in the morning and increasing abdominal girth or tightness of her shirt with bloating that were present in the other patients with IE. Reducing her sodium intake to about 1.5 g/day resulted in less than a 0.6 kg weight gain from morning to bedtime and reducing nocturia to once a night.

The fourth patient was a 23-year-old woman who presented with a recent history of microscopic hematuria with a biopsy diagnosis of a thin basement membrane, gastroparesis, recovery from COVID-19 infection, and signs and symptoms typical of IE along with nocturia × 4 to 5. She weighed 74 kg and gained 2.5 to 3 kg from morning to bedtime on a 3-to-4 g sodium intake along with the nocturia. She was treated with a low sodium diet approximating 1.75 g per day but required hydrochlorothiazide to attain weight gains of less than 0.5 kg from morning to bedtime with absence of nocturia [13].

**Figure 5:**
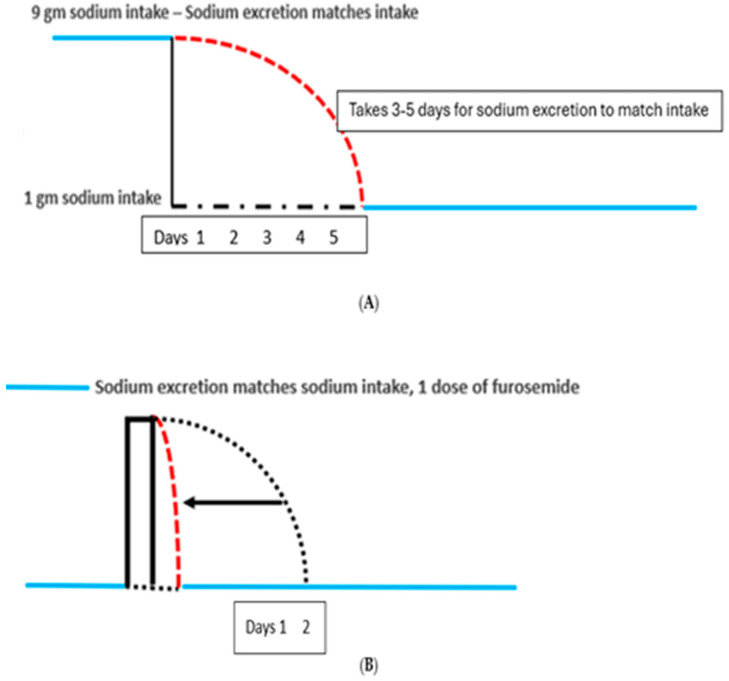
(**A**) Graph showing patient in balance in blue lines where sodium intake matches output. The patient in sodium balance on a sodium intake of 4 g acutely reduces sodium intake to 1 g/day. The red hashed lines represent sodium excretion exceeding sodium intake of 1 g/day. It takes 3–5 days for the body to eliminate the excess sodium before reaching a steady state where input equals output or to be in sodium balance. (**B**) Effect of one episode of increased sodium intake. Furosemide following AM eliminates 3–5 days of bloating. Graph showing a patient in sodium balance on a low salt diet in blue line on lower left. The patient suddenly increases sodium intake by a single high sodium meal which would normally take 3–5 days to excrete as noted by black dotted line before going into sodium balance in blue line. A single dose of furosemide rapidly excreted the excess sodium to place the patient in sodium balance in 1 day in hashed red line and eliminate the sense of bloating and edema.

All four patients had difficulty sustaining consistently low sodium intake because of lifestyle circumstances that prevented them from maintaining a low sodium diet. A single episode of dietary indiscretion led to the discomfort of cyclical weight gains that took 3 to 5 days to resolve after returning to their customary low sodium diets. Any normal subject, who acutely reduces their daily sodium intake, will gradually reduce sodium excretion and reach a new equilibrated state in 3 to 5 days when sodium intake matches sodium excretion, Figure 5A [19].

Reaching equilibrium within 3 to 5 days was found to be true in IE patients after a single episode of eating a diet of higher sodium content. They had experienced truncal and abdominal girth with bloating during that time period. However, the patients who took hydrochlorothiazide (HCTZ) the morning after consuming a high sodium intake the night before were able to reach equilibrium within 1–2 days (Figure 5B).

This was found to be true in patients with IE where they experienced increased truncal and abdominal girth with bloating for 3 to 5 days after a single episode of eating a diet of higher sodium content. The patients took hydrochlorothiazide (HCTZ) the following morning whenever they had a high sodium intake the night before, which eliminated the 3 to 5 days of edema and bloating, Figure 5B [13,14]. HCTZ was switched to 20 mg of furosemide because of the hypokalemia that required potassium supplementation with HCTZ.

## 5. Unusual Life-Threatening Complications of Idiopathic Edema

The majority of IE cases present with the cyclical edema and nocturia as described above. Unusual life-threatening complications can occur when the physiological consequences of the capillary leak are manifested by activation of other metabolic pathways. The first patient was an 81-year-old woman from Florida, who was referred for evaluation of three episodes of seizures and coma due to hyponatremia. It appears that she had symptoms of hyponatremia since the age of 77 when she experienced staggering gait and bumping into objects that bruised or abraded her knees [20,21,22]. In 2020, she was hospitalized for three episodes of seizures and coma with serum sodium of 117, 123, and 123 mmol/L, respectively. We explained that a large intake of water over a short period of time resulted in a rapid decrease in serum sodium which induced swelling of brain cells that was responsible for her coma and seizures. A larger volume of water intake could have led to greater swelling of the brain cells in a rigidly enclosed bony space that would cause sudden death by forcing the brainstem into the spinal canal as described in marathon runners and other sports activities [22,23,24]. One episode resulted in fracture of her sacrum that was associated with a seizure and coma while driving an automobile. She was intubated for extended periods of time which eventually required resection of a 90% stenosis of her trachea. When seen in New York, the limited information presented to us suggested that she might be suffering from a reset osmostat when she was noted to excrete a dilute urine with an osmolality of 158 mOsm/kg when her serum sodium was 133 mmol/L. While considering the rarity of dilute urines being excreted in hyponatremic conditions and an expected lower serum sodium when urines become dilute in reset osmostat [25], the patient mentioned that she gets up four times a night to urinate. Our first manuscript on IE stressed the “unappreciated importance of nocturia” that characterized patients with IE [13]. We proceeded to evaluate her for IE and realized she had swollen hands and face on arising and increased abdominal and truncal girth with bloating after assuming an upright posture that was followed by nocturia × 4 since the age of 18. Weights taken in the morning and at bedtime over 7 days revealed weight gains between 2.5 and 3.1 kg with nocturia × 4 that confirmed the diagnosis of IE. As noted above, the increased capillary leak in IE reduces intravascular volume, which is a potent stimulus for increased ADH production. ADH levels remain high because the inhibitory effect of the hypo-osmolar plasma is overridden by the more potent volume stimulus for ADH secretion [26]. Under these volume-depleted conditions, the hyponatremia will persist as long as the patient is drinking sufficient amounts of water, as normal subjects receiving daily administration of pitressin did not develop hyponatremia without adequate water intake [27,28,29]. The intravascular volume depletion also creates a prerenal state which decreases GFR, urine volume, and solute excretion to promote edema formation when in an upright position, Table 2 [6,30].

When the patient assumes a recumbent position at bedtime, fluid and protein from the interstitial space moves into the intravascular space to normalize or increase intravascular volume which would reduce plasma renin, aldosterone, and ADH levels, increase GFR, and excrete large volumes of dilute urines of high solute content to increase nocturia and return to a non-edematous state in the morning. An overnight collection of urines during her second hyponatremic hospitalization illustrates these points when she excreted a total volume of 3100 mL of urine with osmolalities that became progressively dilute at 251, 197, 165, and 146 mOsm/kg. The excretion of dilute urines removes pure water from the body to increase serum sodium or correct the hyponatremia. The complicated pathophysiologic changes noted during upright and recumbent positions in the hyponatremic IE patient are identical to a hyponatremic patient with renal salt wasting (RSW). The only differences are 1. intravascular volume depletion in RSW is due to urinary losses of solute and water as compared to a shift in solutes and water from intravascular to interstitial spaces due to a capillary leak in IE and 2. correction of intravascular volume depletion and hyponatremia by isotonic saline infusions in RSW and shift of solutes and water from interstitial to intravascular space in IE [14,31]. In both conditions, the restoration of a high or normal intravascular volume eliminates the volume stimulus for ADH secretion and allows the coexisting hypo-osmolality of plasma to inhibit ADH secretion, inducing excretion of large volumes of dilute urine to correct the hyponatremia [11,14,31].

The constant fear of having seizures was addressed by repeatedly explaining how we can eliminate the seizures and coma by avoiding intake of large volumes of water over a short period of time and the increase in truncal and abdominal girth with bloating and nocturia by reducing her sodium intake or add furosemide if she had a high sodium meal the night before. Controlling water intake to 4 ounces of water every 3 h during the waking hours of the day led to serum sodium levels consistently above 137 mmol/L with more recent levels exceeding 139 mmol/L. It was difficult to limit her sodium intake to less than 1 g a day because of very active social engagements. She learned to take furosemide the morning after consuming a high sodium diet the night before to eliminate the 3 to 5 days of edema and bloating, Figure 5A,B [19]. She was repeatedly reminded that the staggering and seizures with coma were due to excessive water intake and her swelling, increase in truncal and abdominal girth with bloating, and nocturia were due to high sodium diets. In the course of two years, all fears of seizures and coma and the complex of symptoms associated with IE dissipated as she is able to travel unsupervised and participate in many social activities. She is no longer fearful of having episodes of seizures and coma and understands why she had such puzzling effects of IE since age 18. She understands the derivation of her symptoms and has greatly improved her physical and emotional well-being.

The second patient is a 42-year-old woman with a history of immunologic deficiency with probable Sjogren’s syndrome and Raynaud’s phenomenon, proteinuria with asymptomatic microscopic hematuria since age 4 and frequent urinary tract infections. She presented with a history of postural dizziness with a single episode of fainting between 2000 and 2004. She had 4 to 5 fainting episodes with intermittent postural dizziness between 2004 and 2010. She fainted 6 to 8 times and fractured her wrist, elbow, shoulder, and coccyx in 2016 and sprained her ankles 4 to 5 times, wrist 2 to 3 times, knee and shoulder that required long periods of rehabilitation, and remained homebound and unable to work. She had foam placed over hardwood floors and any sharp edges, nonslip carpets on the stairways, and about 20 rings placed at strategic areas of the wall to hold on to when she was dizzy while ambulating. She also sat down while showering and had access to a panic button should she fall or faint in the bathroom. Her lying blood pressure of 94/50 decreased to 55–60/25–30 mmHg standing as her pulse increased from 84 beats per minute lying to 136 beats per minute standing, which were more consistent with a volume-depleted state rather than having autonomic failure where there is a meager increase in pulse rates after standing up [32]. While undergoing a diagnostic evaluation of her symptoms, she mentioned getting up four times a night to urinate. The diagnosis of IE was made by the history of swelling of hands and face in the morning followed by an increase in truncal and abdominal girth, bloating, and edema with nocturia. She gained 2.0 to 2.7 kg from morning to bedtime and got up to urinate four times each night for one week. Her weight upon arising in the morning was 63 kg. It was apparent that her capillary leak was of such magnitude that there was sufficient amount of fluid moving from the intravascular to interstitial spaces to reduce blood volume to the extent of inducing severe postural hypotension, dizziness, and fainting. The postural dizziness and fainting associated with IE eliminated salt restriction as a therapeutic option. The most reasonable option was to wear support stockings extending up to her waist to increase interstitial hydrostatic pressure and prevent fluid from leaving the intravascular spaces. On this regimen, her lying blood pressure of 94/57 was unchanged at 95/65 standing while her pulse increased from 88 beats per minute lying to 121 beats per minute standing. The support stocking also eliminated the weight gain from morning to bedtime and nocturia. Her postural hypotension and signs and symptoms of IE were no longer a problem as long as she wore her support hose that extended to her waist. She was fortunately not obese like many of our other patients who would have had great difficulty wearing the support stockings. Postural hypotension of this severity would be extremely difficult to treat in very obese patients but should be attempted because there are no alternatives for this life-threatening complication of IE. There is a single case of a patient who had intermittent bouts of severe capillary leak that led to her demise from one of her recurrent hypotensive episodes [18]. This case does not appear to fall under the category of IE because of the intermittency of the disease, which has not occurred in other patients with IE over extended periods of time. Capillary leak has been demonstrated in many clinical conditions such as sepsis, autoimmune diseases, drugs, and hypoestrogenism that add further insights into clinical outcomes resulting from a capillary leak [33,34,35].

## 6. Summary and Concluding Remarks

It is apparent from the cases included in this review that an increase in capillary leak can lead to seemingly disparate clinical presentations. We are uncertain of the prevalence of IE in the general population, but it appears to be more common than it is perceived to be. The duration of undiagnosed cases of IE included in this review suggests that IE does not appear to be readily recognized as a disease. Recognizing and understanding the spectrum of pathophysiologic and clinical presentations resulting from a single leaky capillary membrane greatly improved the physical and emotional well-being of these patients. Understanding led to substantial emotional stability. Treatment of IE can be confusing but as discussed above, because a low sodium diet that eliminates the distressing signs and symptoms of IE requires much effort to achieve, the option to take 20 mg of furosemide every morning while continuing their usual higher sodium diets can greatly simplify and improve quality of life for these patients. While we advocate an attempt to understanding and teaching the pathophysiologic consequences of a capillary leak, a practical clinical approach can be summarized by highlighting the sequence of events that will lead to a diagnosis and treatment of these patients, Table 3.

## Figures and Tables

**Table 1:** Criteria used to make the diagnosis of idiopathic edema, according to Thorn and McKendry.

**Thorn Criteria**	
	Weight gain ≥ 1.4 kg between the morning and night measurement
	Active exclusion of organic diseases that predispose to edema formation
	Evidence of substantial psychological or emotional disturbance
**McKendry criteria**	
	Non-pitting edema of face, trunk, or upper limbs unrelated to menses
	Weight gain between 8 AM-8 PM of 2 or more pounds on at least one third of the days
	Weight gain in 1 day of 4 or more pounds unrelated to menses
	Worsening or onset of nervous tension, irritability, headache in phase with edema
	History of menstrual dysfunction
	Personal history of diabetes mellitus, big babies, glycosuria, function hypoglycemia or repeated abortions
	Family history of diabetes or big babies (9 pounds or more)
	Nervous temperament or autonomic instability
	Overweight
	Onset age 20–60 years

**Table 2:** Clinical and laboratory presentation of patients with idiopathic edema.

**Upright Position**			
	Increased intravascular hydrostatic pressure		
	Fluid and proteins leave the intravascular space through “holes” in the vascular membrane		
	Edema formation		
	Resulting in	**Decreased**	**Increased**
		Intravascular volume	Renin
		GFR	Aldosterone
		Urine volume	ADH
		Solute excretion	Morning to night body weight > 1.4 kg
**Recumbent Position—“a reversal of events”**			
	Fluid and proteins now move from the interstitium back to the intravascular space via “holes”		
	Resulting in	**Increased**	**Decreased**
		Intravascular volume	Renin
		GFR	Aldosterone
		Urine volume	ADH
		Solute excretion	Edema
		Nocturia: leading to edema-free weight in AM	

**Table 3:** Diagnostic and therapeutic considerations for Idiopathic Edema.

**Signs and symptoms**	
	Patient is a woman
	Nocturia
	Varying degrees of swelling in thorax, abdomen, legs after standing upright
	Greater than 1.4 kg. (circa 3.1 lbs.) weight gain from awakening to bedtime for 1 week
	Postural hypotension—if present in any woman, consider IE
**Hyponatremia**	
	Staggering, difficulty walking, falling, altered mentation, rarely seizures
	When severe capillary leak is present, patients may develop seizures or coma
	Serum sodium usually < 135 mEq/L.
**Treatment**	
	Sodium restricted diet [which is contraindicated in those with postural hypotension]
	Single dose of a diuretic, especially after a large, single increase in sodium intake
	Daily dose of a diuretic in patients exposed to daily large sodium intake
	Avoid ingesting large volumes of water or other salt free fluids
	Support stockings to high waistline for patients with postural hypotension

## Data Availability

No new data was created or analyzed in this study. Data sharing is not applicable to this article.

## Abbreviations

ADH	antidiuretic hormone
DOCA	deoxycorticosterone acetate
CHF	congestive heart failure
GFR	glomerular filtration rate
HCTZ	hydrochlorothiazide
IE	idiopathic edema
PCOS	polycystic ovary syndrome
RSW	renal salt wasting
